# Systematic Review of the Longitudinal Sensitivity of Precision Tasks in Visual Working Memory

**DOI:** 10.3390/vision6010007

**Published:** 2022-01-21

**Authors:** James Ades, Jyoti Mishra

**Affiliations:** Department of Psychiatry, School of Medicine, University of California San Diego, La Jolla, CA 92093, USA; Jymishra@health.ucsd.edu

**Keywords:** precision, resolution, longitudinal, visual, working memory, reproduction, change detection

## Abstract

Precision of working memory (WM) refers to the objective performance of individuals when trying to recall the features of the encoded WM items. Studies of precision in VWM aim to identify whether differences in WM performance within individuals are sensitive to individual states or traits. In this systematic review, we study VWM precision and whether it reflects true differences in ability to accurately store information, and thereby possibly a more sensitive measure than discrete VWM span alone. Sifting through 327 abstracts, we identified 34 relevant articles. After assessing these articles with regard to our inclusion criteria to test participants at two separate time points and have a sample size of at least fifteen participants, we found four longitudinal studies regarding VWM precision. One review author and two reviewers independently assessed all studies in the screening and selection process and extracted outcome measures, study characteristics, and, when possible, test–retest reliability metrics. Given the small and heterogeneous sample, this systematic review could not yet provide conclusive evidence on the sensitivity of VWM precision paradigms. Future research of VWM should include longitudinal studies of precision, and address both test–retest reliability in healthy adults and changes in precision during key developmental trajectory periods and in clinical populations.

## 1. Introduction

Visual working memory (VWM) comprises three components: encoding, maintenance or manipulation of that information over a short duration, and retrieval. Highlighting the overall importance of studying WM, research shows a strong correlation between WM and fluid reasoning, which itself has been linked to academic and career success [1,2,3]. VWM is fundamental to cognitive performance, and limitations in WM capacity are reliably associated with individual variance in a range of cognitive functions such as reasoning, skill acquisition, processing information, and comprehension [1,4]. In these studies, WM capacity refers to span, defined as the maximum number of items that an individual can encode and maintain, for a short period of time generally greater than 1.5 s.

WM capacity deficits bedevil clinical populations including Parkinson’s disease, autism, schizophrenia, and attention deficit hyperactivity disorder (ADHD) [5,6,7,8,9]; hence, it is an important and clinically meaningful measure of WM. Yet, WM capacity derived from a span measure provides only discrete information, and therefore, lacks nuance. Usually from span tasks, we can only derive whether the information recalled matched the originally encoded information. The degree to which the recalled information differs from the encoded information (recall performance), or the consistency of recall across trials (recall precision), is not usually tracked. On the other hand, in continuous report studies, the retrieval phase of WM demands participants to reproduce the exact items they encode. Recent reproduction studies incorporating precision or resolution (i.e., individual capability to precisely report a feature of the encoded information such as orientation) have shown promise in discerning subtle changes in VWM capacity within individuals across the lifespan, as well as across clinical populations [9,10,11]. These reproduction studies suggest there may be an ideal VWM metric that sufficiently quantifies and contextualizes important differences between and within participants over time. Thus, this systematic review aims to determine the validity of VWM precision (consistency of recall, i.e., the more variable the responses the less precise the recall) as a more sensitive measure of VWM capacity than discrete item count, using exclusively longitudinal studies. 

Humanity’s progression has been accompanied by its scientific advancement in measuring that progression: the milliseconds Usain Bolt bests the world record by in the 100-meter dash, the inches Michael Jordan outleaps his opponents, the nanometers in a carbon nanotube, and the number of genes in the human genome. Constructing a VWM measure more nuanced than a discrete one is important for five reasons: (1) to better understand developmental trajectories and associated behaviors; (2) to more tactfully diagnose cognitive dysfunction; (3) to rigorously evaluate the effects of cognitive intervention programs; (4) to establish the efficacy of treatments affecting cognition among clinical populations; and (5) to employ it as a potential motivating force to enhance participant engagement. We offer the above not as the objectives we aim to validate here, but as motivation for this work, in hopes to push the field toward such a nuanced metric.

Systematic reviews aim to minimize bias through developing a research question and employing guidelines (we use PRISMA’s: [12] to systematically (through criteria) cull all studies within a defined time period relevant to the research objective. Hence, the research objective, the search process, and the inclusion criteria ultimately determine what studies are included in the review. The first, and arguably most important, part of this process is to generate a PICOS (participants, interventions, comparators, outcomes, and study designs) question. Our PICOS question is: in looking at intraindividual differences in individuals, in either longitudinal studies of developmental and/or elderly populations or clinical populations measured at two timepoints, can we identify a reliable VWM measure that is more precise than discrete capacity limits? Of note, in our search, we found that precision, resolution, fidelity, and sensitivity are all used somewhat interchangeably in the literature, and generally connote the extent to which one can capture or encode stimulus features (here, though, we distinguish sensitivity as the task’s potential to extract information regarding precision). It is worth noting that the international organization for standardization refers to precision (ISO 5725 Accuracy) as a measure of scatter, usually expressed as imprecision and computed as the standard deviation of the test results. We also found that besides one study of motor learning and working memory in children born preterm [13], in the entire VWM field, there have been no systematic reviews conducted within the field of VWM.

While cross-sectional VWM studies of precision abound, these single timepoint studies not only fail to address the elemental test–retest reliability of the paradigm but also the fundamentally important and often ignored role of within-subject growth. Hence, we focused only on those studies with repeat VWM sessions. In our systematic search process, we retrieved few longitudinal studies of VWM precision. Thus, the conclusion of our work is an important (and urgent) if uninteresting one: the lamentable dearth of longitudinal studies in VWM precision prevents us from concluding whether it is a more sensitive assessment than VWM span. Still, through this systematic review, we provide an important direction for the field moving forward. 

## 2. Methodology

### 2.1. Eligibility Criteria

We excluded non-data-based studies—reviews, research protocol, editorials, letters—as well as articles not published in English.

Our inclusion criteria for this systematic review required that studies tested participants at two separate time points and that the sample size of these studies exceeds 15 participants. We did not consider age for this review. To minimize publication bias, we did not require that studies be published.

### 2.2. Information Sources and Search

We selected five electronic databases for our search (PubMed, PsychINFO, Embase, Cochrane Register of Controlled Trials, Web of Science). Only articles written after 1 January 2000 and before 31 September 2018 were considered for this review. Subject headings and keywords were specific to each database and are listed in Table 1, included in the Section 3. We used citations and references in relevant articles to ensure the inclusion of every pertinent longitudinal study; we employed all references in the bibliography of articles selected for this review; and we sought unpublished research through Clinicaltrials.gov (accessed on 22 October 2018), grey literature at Scirus.com (accessed on 23 October 2018), and Opengrey (formerly System for Information on Grey Literature in Europe). The search was conducted by the first author. 

### 2.3. Study Selection

Two research staff served as independent reviewers and participated in the screening and selection process. Using the open-source software, Zotero, JA sorted all abstracts and articles into two overarching categories: longitudinal and cross-sectional. Within each of these categories, there were three subcategories: continuous recall, change detection, and other. Four separate categories were made for articles that did not fall under any of these particular categories; these categories were “Articles that cannot be accessed”, “Duplicates”, “Questions”, and “Retracted”.

JA duplicated all abstracts from the five databases and then collated them into one folder, such that no reviewers knew the database source. JA instructed the two reviewers to place studies that seemed ambivalent into the “Questions” folder. 

We located all articles with abstracts that did not overtly mention VWM task methodology. The only article that could not be accessed was also one that was retracted. If a study was longitudinal, we assumed that this would be stated in the abstract itself. We later discussed and reallocated any abstracts in the “Questions” folder. 

Studies were reviewed in the same manner as the abstracts, with JA independently reading through all articles, and the two independent researchers splitting the articles and deciding whether articles met inclusion criteria.

### 2.4. Data Collection Processes

JA used an extraction sheet to gather relevant information from each study. Other reviewers checked the extracted data. Disagreements were resolved by discussion between JA and other reviewers. Any unresolved disagreements were then forwarded to a third independent reviewer. 

In total, we found four research articles and one unpublished study that met criteria for inclusion.

We followed-up via email with authors of all selected articles when necessary information could not be located in the manuscript. All authors we contacted responded. Each supplied the supplementary information except in the case of Zokaie et al. [9], where the authors were constrained by the ethics section of the NHS study, requiring approval prior to study initiation for the sharing of raw anonymized data.

### 2.5. Data Items

(1) We extracted participants’ age, mental health status, outcome measures of recall performance and precision, and in the case of noncontinuous tasks, mean performance. Regarding clinical mental health status, we did not record severity of disorder or medication dose (these variables were not relevant to our PICOS question).

(2) From the longitudinal studies, we extracted sample size and attrition rates. In addition, we collected duration intervals between testing timepoints, the reproduction and control task(s) used, and the statistical analyses employed by each author.

(3) Additionally, we sought test–retest reliability metrics from studies with healthy controls conducted over test–retest periods of a short duration, which Trevathan [14] defines as time which is large enough that respondents are not likely to remember or be influenced by their first set of responses when providing their second set, but small enough that genuine differences in scores are not likely to have occurred.

We made several assumptions. (1) Precision of VWM is measurable in human participants. (2) Given no stated mental disorder among healthy control participants, there exist no latent cognitive issues. (3) Effects of medication (i.e., on vs. off), such as in Parkinson’s Disease (PD) patients, are statistically comparable to the effects of time for healthy controls. (4) Participants were provided comparatively similar testing environments.

### 2.6. Risk of Bias in Individual Studies

For all included studies, we assessed sample size (of both healthy control and clinical samples, where such groups existed), effect size calculations, participant retention, ancillary tasks, and potential biases in analysis (such as assessor blinding). We also report whether analyses were preregistered. 

### 2.7. Summary Measures

Though authors use different terminology for performance outcomes in the selected studies, most continuous recall studies provide measures of two variables: recall performance and precision. Recall precision is measured as the inverse variability in response around the probed target feature (1/SD), though in both Burnett Heyes et al. [10] and Zokaie et al. [9], sensorimotor error is also taken into account, such that precision is calculated as 1/square root (variance (WM error)—variance (sensorimotor error)). The more variable the responses, the less precise the recall (Fallon et al. provide kappa values vs. recall precision, which correlate significantly with recall precision). This is different from other measures, where reliability is defined as the ratio of the difference between observed variance and mean of squared standard errors relative to observed variance.

On the other hand, recall performance (mean angular error) corresponds to the proximity of a participant’s reproduction of an item feature to the actual item feature. The closer one’s answer to the original orientation of the item, the greater the recall performance. Given that the forced-choice Adam and Vogel [15] task measured mean performance, we ultimately used a difference in mean effect sizes to compare and assess changes in precision over time. Additionally, we used Pearson’s correlation coefficient to look at test–retest reliability.

### 2.8. Synthesis of Results

Study design, participants, and reported outcomes were not sufficiently similar to warrant the combination of data into a meta-analysis. Furthermore, there were too few studies. Per PRISMA’s recommendation, we thus focused on “describing the studies, their results, their applicability, and their limitations and on qualitative synthesis rather than meta-analysis’’ [12].

### 2.9. Risk of Bias across Studies

We reduced biases across studies in several ways. First, in our study search strategy, we reduced information bias (missing relevant studies from a narrowed search scope) by prioritizing search recall. Here, search recall refers to the number of articles retrieved in a search, while search precision refers to a constraining of search parameters to exclude irrelevant results. 

Second, we further minimized publication bias (i.e., the problem that valid studies with nonsignificant findings are less likely to be published than those with significant results) by searching two databases of registered studies: Cochrane Register of Studies (formerly, Cochrane Central Register of Controlled Trials) and Clinicaltrials.gov. Furthermore, as previously mentioned, we also searched grey literature through Opengrey (formerly System for Information on Grey Literature in Europe).

Third, we lessened selective bias (reporting statistically significant and interesting results while ignoring those that are not) by investigating—and reporting—whether or not trials were preregistered and whether (and the extent to which) the protocol differed from the published articles.

## 3. Results

### 3.1. Study Selection

Table 1 documents the total abstracts retrieved from each of the five databases and the corresponding search terminology we used.

**Figure 1:**
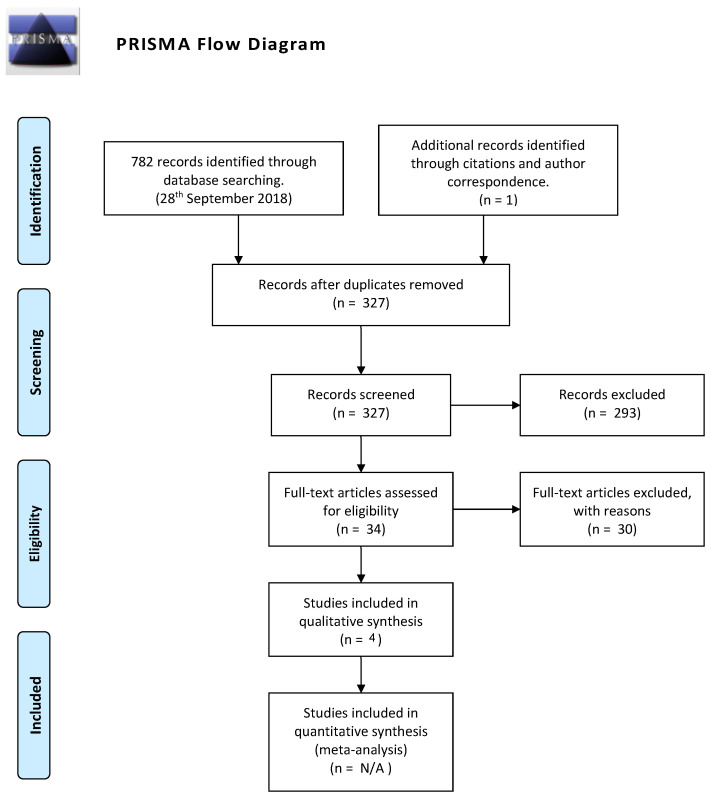
PRISMA flow diagram of study selection process.

The PRISMA flow diagram (Figure 1) documents the systematic process of our search and the exclusion of articles.

In total, our search returned 782 results. These abstracts were imported into Zotero; we removed duplicates using Zotero’s duplicates feature. Three of the five additional duplicates were author-initiated retractions and were found by reading through abstracts. We winnowed the number of distinct abstracts to 392 (including one additional article found through citations: “Developmental and individual differences in the precision of visuospatial memory”). After a primary screening of these abstracts, we determined that 34 abstracts warranted full article review. Of these, we determined that four articles and one study met our inclusion criteria. One unpublished study from Project iLEAD [16] was not included because of problems with the implementation of an adaptive algorithm, warranting its exclusion. 

### 3.2. Study Characteristics 

Here, we enumerate study characteristics within each of the four included studies. Study characteristics are categorized in Table 2. In addition, the main task paradigms from these studies are illustrated in Supplementary Figure S2. The first three studies employ a continuous recall task; the fourth study is a whole-report, forced-choice task. 

Zokaei et al. [9] assessed 114 healthy individuals (comprising children, young adults, and older adults, ages 9–80 years; 18 female and 97 male), as well as 12 PD patients (ages 51–79 years; six female, six male) beginning treatment. Healthy individuals completed only one session; PD patients were tested prior to medication and then three months later, having established treatment. Hence, only PD patients were longitudinally tested. All participants received a pre-cueing task, controlling for the participant’s ability to maintain relevant stimuli and filter nonrelevant information. In this task, participants were instructed to remember the first item despite the presentation of three subsequent items. All healthy control participants completed 200 trials; PD patients completed anywhere from 100–200 trials. In addition, all PD patients and a subset of healthy controls (*n* = 10) received a sensorimotor task (25 trials). This sensorimotor task controlled for dexterity and motor precision (participants turned a dial to reorient the probe bar to match the orientation of a simultaneously presented target item). All participants received both one-item and four-item continuous recall tasks. In each task, participants were shown a colored, randomly oriented bar. The target item disappeared, and following a 500 ms delay (blank screen), participants were either cued with a probed bar (if conducting the one-item task) or presented with additional colored bars sequentially delivered with a 500 ms delay (in this multi-item condition, participants were then presented with a horizontal bar with the color of the corresponding target item from the sequence and participants used the dial to rotate the bar to the correct orientation of the colored target item). Children (ages 9–13.5) completed 90 trials of the three-item task and adults completed 200 trials of a four-item task. All participants completed a digit span task. Healthy older participants (ages 53–80) and PD patients also completed a Corsi-spatial span task. 

Burnett Heyes et al. [10] measured VWM at two timepoints, separated by two years. In their forty-child (ages 7–13; 40 male) sample, children received four tasks, three of which—sensorimotor, one- and three-item sequential recall—were identical to those used by Zokaei et al. [9]. Children also completed a color-naming task, testing for color-blindness. Taking a break every 15 trials, children completed 30 trials of the one-item task (divided into two blocks of 15 trials) and 90 trials of the three-item task. All colors and serial positions were probed with equal probability. In addition, the Full-Scale Intelligence Quotient (FSIQ) test was also administered.

Fallon et al. [17] studied 20 patients diagnosed with PD, currently on dopaminergic treatment (age: mean 65.7; 9 female, 10 male). Further, 17 healthy older adults served as a control (age: mean 68.5; eight female, nine male). All participants completed two sessions within 1 to 4 weeks. Medication for Parkinson’s patients was reversed such that patients taking medication for initial testing were off medication for the following session and vice versa. At both sessions, healthy older adults completed 64 trials of four conditions (totaling 256 trials); PD patients completed 128 trials. The four conditions were “ignore”, “maintain”, “update”, and prolonged maintain. In the ignore condition, participants held relevant information while simultaneously contending with distracting stimuli. The first maintain condition functioned as a temporal control for the ignore condition: participants were not presented with any distracting stimuli and, instead, they were to remember both items for two seconds. In the update condition, participants were presented with a new pair of stimuli and had to supplant these stimuli with the first stimuli. The final maintain condition served as a temporal control for the update condition: it lasted six seconds—the same total duration as the update condition. Participants had to maintain both stimuli for the full length of time.

Adam and Vogel’s study [15] comprised four conditions, focused on performance feedback in VWM tasks, and was inherently different from the previous three studies. They collected 72 healthy subjects (ages 18–35 years; 48 female, 24 male). Participants were split into three pseudorandom training groups (a fourth, passive control group was later added). Over a four-month period flanked by pre- and post-tests, two of the groups practiced VWM tasks, the other two did not. The pre- post-test tasks included three VWM tasks: a color whole-report task, a color change-detection task, and an orientation whole- report task. Only the orientation whole-report task is relevant to this review. In this task, participants were provided targets in the same array (whole report) and were then spatially cued on one of those targets.

The nonincluded study mentioned in the study selection section was a change-detection task employing an adaptivity paradigm that imitated the filter task (Vogel et al., 2005).

### 3.3. Results of Individual Studies

First, we looked at changes in recall precision over time and then recall performance. Table 3 summarizes the results of the individual studies.

Recall precision (calculated as the reciprocal of the standard deviation of error across trials) represents the degree to which the participant can reproduce the stored items. Both Zokaei et al. (2015) and Burnett Heyes et al. [10] found a correlation of sensorimotor performance with age, and corrected for this in their recall-precision calculations. In Zokaie et al., three months after starting treatment, PD patients realized significant increases in precision, t(11) = 3.01, *p* = 0.012. Using the nonparametric Wilcoxon signed-rank test (precision data in this study were not normally distributed), Burnett Heyes et al. [10] compared developmental changes and found that VWM precision in children increased for both one-item (Z = 2.87, *p* = 0.004) and three-item (Z = 2.39, *p* = 0.017) conditions over the two-year period (See Supplementary Figure S1). Furthermore, comparing changes in VWM precision between one- and three-item tasks, Burnett Heyes [10] found a steeper developmental trajectory of the three-item task (Z = 2.79, *p* = 0.005). Interestingly though, while a comparison between t1 vs. t2 at each serial position of the three-item target revealed significant findings for the first and second items (Z = 2.05, *p* = 0.04 and Z = 3.06, *p* = 0.002, respectively), there was no significant difference for the final item (Z = 1.57 *p* = 0.12). Fallon et al. (2017), who employed kappa (concentration parameter), which measures the concentration of responses around the target through a probabilistic model and highly correlates with recall precision (higher kappa equals less variability), also used a Wilcoxon signed-rank test to analyze change in kappa. While patients in this study had significantly lower kappa values for longer, compared with shorter, retention trials (Z = 3.26, *p* = 0.001), this duration effect was not significant in controls (Z = 1.39, *p* = 0.163). Looking at the effects of duration on kappa values between participant groups, Fallon et al. (2017) found that patients were significantly more affected by duration (Z = 2.61, *p* = 0.009). 

Recall performance (mean angular error) is the absolute angular difference between target and response orientations. Postmedication, Zokaie et al. [9] found significant improvements in PD patients’ performance compared to premedication across all tested targets (main effect of medication, F(1,11) = 9.08, *p* = 0.012; main effect of serial position of sequential target, F(3,33) = 12.6, *p* < 0.001; no significant interaction between medication and serial position: F(3,33) = 2.5, *p* = 0.07). Incorporating the probabilistic mixture model [18], Burnett Heyes et al. found that in the three-item task, the proportion of responses near the target orientation increased from t1 to t2 (the distribution of responses narrowed with a lower proportion of responses at the tail of the distribution; see supplementary Figure S1). All other model parameters remained the same (unchanged sources of error) t(39) = 1.6, *p* = 0.12 for *p*(T), t(39) = 1.4, *p* = 0.18 for *p*(NT) and t(39) = 0.3, *p* = 0.73 for *p*(U). Here, *p*(T) refers to proportion of target responses, *p*(NT) refers to the proportion of nontarget responses, and *p*(U) refers to random responses. Thus, Burnett Heyes et al. located performance improvement in precision. Contrary to Zokaei et al., Fallon et al. [17] did not find a main effect of medication in the PD sample, and thus, the two sessions were collapsed among patients and controls. Notably, the PD patients in Zokaei et al. were newly diagnosed and had not yet begun treatment, while Fallon et al. studied PD patients on stable dopaminergic medication with near perfect Montreal Cognitive Assessment (MOCA) scores, suggesting no cognitive dysfunction. Regardless of whether they were in the PD or healthy control group, participants in Fallon et al. fared significantly worse on updating and ignoring trials compared with maintain-only trials (F(1,36) = 18.41, *p* < 0.001). Participants’ recall was further impaired by longer retention intervals.

Other measures: The primary focus of the Adam and Vogel [15] study considered the effects of feedback on VWM training, and thus, the authors predominantly investigate the interactions between groups and individual practice sessions, rather than detailing the differences between pre- vs. post-test results. Though these results are graphed, they are not overtly statistically analyzed. Adam and Vogel do state that they looked separately at each group for evidence of improvement from pre- to post- test. Oddly, they found only improvement in the no-contact control group (*p* = 0.001). The study data are available on the OSF site (Adam K.C.S. & Vogel E.K., 2018), and we analyze data for the whole-report orientation task using two-tailed *t*-tests for pre- post- test results. We calculated whether there was an observable increase in precision at postintervention for those who received VWM training (albeit with a whole-report color task, not the orientation one), as this would seem to be the only conceivable reason for VWM improvement on the orientation task. Our finding was odd: the combined first two groups receiving VWM training did not improve on the orientation task (t(47) = −1.68, *p* = 0.1), but the combined control (those who received either a crossword puzzle or no contact) did (t(52) = −2.01, *p* = 0.05). Indeed, when we used the latter group (*n* = 53) as a control for test–retest reliability (as this group received no training and would have no plausible reason for VWM change), the Pearson’s correlation coefficient for the whole report was 0.80 (*p* < 0.005). 

Additionally, as stated above, we calculated test–retest reliability in the Fallon et al. study. The Pearson’s correlation coefficient of healthy controls (*n* = 17) for the mean angular error for each condition (update, ignore, maintain 2000 ms, maintain 6000 ms) were 0.36, 0.50, 0.53, 0.75, respectively; for kappa values (concentration parameter and highly correlated with precision), they were 0.37, 0.48, 0.53, 0.57. 

### 3.4. Synthesis of Results

While we do calculate standardized effect sizes where possible (Table 4), the studies were so few and the task designs so different (heterogeneity) that we chose *not* to combine participants to perform a meta-analysis. Furthermore, the Zokaie et al. [9] study had fewer than 15 participants in the longitudinal patient-only condition; thus, it would have been unprincipled to combine that study with other results in a quantitative analysis. Instead, we have focused on detailing the characteristics of these studies, their results, applicability, and limitations. Here, we synthesize our qualitative findings

We calculated standardized effect sizes for all but Zokaie et al. [9], which did not include SD or whether Parkinson’s patient data followed a normal distribution. We sought to establish whether precision is a meaningful indicator of VWM sensitivity. Burnett Heyes et al., [10], demonstrated that VWM precision improved with age (7- to 13-year-old children, effect size for two-year change = 0.454) and that this precision further accounted for the corresponding increase in recall performance (given that other error sources remain unchanged). Similarly, Zokaie et al. [9] demonstrated both an enhancement in VWM precision and performance in PD patients who had recently established treatment. On the other hand, Fallon et al. [17] did not find a VWM increase in precision and performance among PD patients on vs. off medication. The results from the Adam and Vogel study did not reveal improvements in precision for those who received feedback, a finding made more significant by the anomaly that those who received no training did improve (t(52) = −2.01, *p* = 0.05)

### 3.5. Risk of Bias across Studies

Our grey literature search did not reveal additional longitudinal VWM studies. We implemented a thorough search strategy to locate published and unpublished longitudinal VWM studies incorporating precision tasks. In addition, we investigated whether and which studies were preregistered to reduce selective bias.

Looking at selective reporting within studies, we found that not all outcome measures were reported. In Zokaei et al. [9], measures for the one-item task were not reported (it was only reported that this measure was used to control for the three-item task). Burnett Heyes et al. [10] did not overtly report recall performance (rather, it was presented in a graph overlaid with the distribution of responses around the target orientation for both timepoints). These authors then used a mixed model to demonstrate the non-significance of other potential sources of error, and reaffirmed that precision accounts for the overall change in performance. However, we know neither the baseline performance nor the magnitude of change in performance.

### 3.6. Risk of Bias within Studies

Table 4 presents the risk of bias within studies based, collating the various study criteria that could affect the interpretation of outcome measures.

### 3.7. Additional Analysis

We computed all test statistics to standardize effect sizes. In cases where the normality of the distribution was provided but the standard deviation was not, we computed effect sizes using the test statistic itself [19,20].

## 4. Discussion

### 4.1. Summary of Evidence

We investigated the main outcomes of recall precision and performance in continuous recall tasks and mean performance in the forced-choice task. Two of these studies found that VWM precision and performance improved with either age (in children/adolescents) or medication in PD patients; one of these studies even found increases in one-item precision. One of these studies found that medication only increased performance in trials where participants were required to update or ignore information, but not when they merely had to maintain it [17]. Lastly, Adam and Vogel found the inexplicable anomaly that participants who received no training improved in mean orientation performance, while those who received training did not (measures of test–retest reliability would certainly help to understand anomalous behavior).

Given the dearth of longitudinal studies observing VWM using precision tasks and the heterogeneity of these tasks, we feel that there is insufficient evidence to conclusively determine whether VWM precision tasks provide a reliable measure of VWM. Only three studies incorporated a continuous recall (production) task, and only two of these were sequential.

We further discuss test–retest reliability in the limitations and conclusion; additionally, though, we would like to see replicability of results from these studies. Replicability will give us a better understanding of the component mechanisms involved. 

Outcomes, if replicable, would have potential relevance for clinical populations (specifically, as shown here, for Parkinson’s patients) and for improved VWM testing in healthy populations. As we state in the introduction, more precise VWM measurements could impact several areas of research: increasing understanding of developmental trajectories and associated behaviors; more discriminate diagnosing of cognitive dysfunction; more precise evaluation of cognitive intervention programs and treatments affecting cognition among clinical populations; and expanding participant engagement, deploying sensitivity as a potentially motivating force. While we hoped to offer conclusions at the outset of this systematic review, given the paucity of quantitative homogeneous research in this field, we instead hope that this collation fortifies the necessity of longitudinal research in VWM precision. In our conclusion, we offer future areas for research that would provide the evidence to sufficiently determine the sensitivity of VWM tasks.

### 4.2. Limitations

This study has several limitations. First, we address limitations at the outcome level, then we address them at the study and review levels.

Production tasks are a comparatively new paradigm in the study of VWM. If medical subject heading (MeSH) terms (and controlled vocabulary from other databases) for VWM were used by databases at all, they served little function. Terms were so broad that they themselves encapsulated VWM. As stated in our methods, we used Boolean logic to curate our results and account for synonyms. In addition, we limited our search to articles in the English language. Still, we ended our review with only four studies, too few and too varied in their outcome measures to move forward with a meta-analysis.

More work needs to be conducted to understand the effects of demographic variables such as socioeconomic status, disease, age, and VWM capacity on precision. Ideally, a range of ages from both healthy (for test–retest reliability) and clinical populations would exist. Regarding Zokaei et al. [9], had all participants at T1 also been tested at T2, we could have collected deeper insight into the longitudinal changes in VWM precision. In Burnett Heyes et al. [10], participants drastically dropped between the two timepoints (56% fewer students at t2).

One major limitation of the included studies is that there exist no test–retest reliability data of these tasks. In the entire VWM field, there has been astoundingly little research into the reliability of VWM tasks. Dai et al. point out in their 2019 paper that “whether VWM capacity estimation is reliable across tests remains essentially unknown.” While both Zokaie et al. [9] and Burnett Heyes et al. [10] correct for changes in sensorimotor precision, this does not replace the specific study of test–retest reliability. While we calculated measures for the Fallon et al. [17] study, a continuous recall task, that task was different enough that we do not feel that those results offer insight into a sequential, single-probe, three-item task. Furthermore, the befuddling improvement (ages 15–35) in those who received no training might be explained by [21] who state that VWM continues to develop through adolescence (even among 16-year-olds); still, this would not explain differences between the group that received training but did not improve. It also might suggest that Pearson’s correlation is insufficient and intraclass correlation should be used (which we did not use for Adam and Vogel because Fallon provided us with Pearson’s coefficients. There is, however, no unanimous agreement on the best metric for test–retest reliability [20,22,23,24].

To date, there are no longitudinal studies of continuous reproduction that manipulate features other than orientation (for instance, color hue). Some cross-sectional studies have found that spatial location, orientation, and color suggest different capacity limits among these features [25,26,27,28,29,30]. We do not know whether precision tasks from one feature would readily translate to another (with regard to sensitivity of improvement) in a longitudinal setting. Importantly, in a recent study looking at psychophysical scaling components of VWM [2,31,32] implement signal detection to argue that the color-wheel task does not sufficiently discern precision, given that color does not subjectively manifest equidistantly in a 360-degree spectrum. Instead, they argue that “memory strength” is the best measure of VWM.

With regard to task design, Fallon et al. limit items to no more than two in an array, a low working-memory load as per the literature [2,31,33,34,35]. Interactions between precision and load need to be determined; some literature suggests precision declines as the number of items (and load) increases [18,36,37,38,39,40]. Others suggest that the number of items will have little effect on precision [41,42,43,44,45,46,47] (designed with a Parkinson’s patient in mind, Fallon et al. focus on ignore and update mechanisms).

While Burnett Heyes [10] found significant effects for the first two sequential items, no significant effect exists for the third and final item. The recency effect, the tendency for participants to more prominently recall the last item presented, has been documented in sequential tasks [48,49,50,51,52], and results suggest that it likely occurred in this continuous recall task as well. Likewise, it is possible that the primacy effect, the tendency to remember the first item, could also explain the large increase in participant performance on the second item. Sandwiched in between the first and third items (both influenced to varying degrees by the respective primacy and recency effects), the second item would be the most difficult to recall, and thus, would also have the largest room to show enhanced performance at retesting. Additionally, it is also possible that interference effects among previously encoded items could explain the performance increase in the second item. In one cross-sectional study, Rudkin et al. [53] demonstrated significant interference between vigilance tasks and sequential visuospatial tasks, an effect that did not occur with a simultaneous visuospatial task. In a similar study of concurrent load and privileged storage, Allen et al. [48] concluded that sequential tasks rely on executive processes to maintain previously encountered stimuli. A final explanation in the case of the child/adolescent studies—separate from an interaction of all of these—could be that with age, students (early adolescents) became more adept at retrieving information (hence the observed increase among the first two items).

Perhaps this suggests, as Zokaei notes, that the continuous recall task burdens VWM with more than mere storage, imposing additional cognitive mechanism(s) in retrieving and/or updating bound stimuli, one possible explanation of Zokaei’s correlative finding between backwards span and the continuous recall task. Still, this demonstrates the concurrent lack of clarity in the additional cognitive costs imposed by the Zokaei et al. and Burnett Heyes et al. sequential tasks; while it might be a prime venue for exploring additional cognitive strain from manipulation and retrieval, the task also lacks the singular capacity to adequately disentangle other processes involved in the storage and maintenance of high-resolution information. If different mechanisms are loaded, beyond pure “storage” of information, this would negate comparability among continuous recall tasks and other precision tasks. Still, such a VWM task with loading several mechanisms might more accurately capture the everyday functional capacities of visual working memory.

### 4.3. Conclusions

Cross-sectional VWM studies show age-associated development through childhood and adolescence [51,54,55], as do executive function tasks with a VWM component [51,56,57]. Guillory et al. [58] and Simmering et al. [59], implementing psychophysical tests in ages 3–8, found that VWM precision developmentally increases through these ages.

Regrettably, this systematic undertaking revealed that most VWM resolution research exists in cross-sectional studies, failing to quantify intraindividual changes over time. Frustratingly, our methodical search also uncovered (in other longitudinal studies failing to meet our criteria) a range of tasks—n-back, span, visual pattern, change detection, etc., exposing perhaps an arbitrariness in VWM task selection. 

Logical next steps include an emphasis on longitudinal studies, specifically studying adolescent, clinical, and aging populations. These studies should incorporate a mélange of forced-choice, change-detection, and production paradigm tasks, to provide both within-subject and between-task comparisons. It is unlikely that healthy, cognitively developed adults will reveal sensitive changes in VWM; however, this population provides a boon to quantifying test–retest reliability, another important aspect of VWM still lacking. We recommend that future studies rely on methods from the above papers, incorporating continuous reproduction from angular rotation. Such studies would help to validate reproduction tasks and better understand the relationship between item span and precision in VWM. Outcome measures should include recall precision and performance.

Furthermore, as one reviewer noted, approximate synonyms beleaguer the VWM field. As we strive for more precise measures of visual working memory, we must also strive for fewer terms and more precise terminology.

Additionally, we must better understand the test–retest reliability of VWM precision within these forced-choice, change-detection, and production task paradigms; and specifically, whether one has advantages over the other. Besides the test–retest reliability calculated from Fallon et al. [17] and Adam & Vogel, there are [15] no measures of test–retest reliability

Worthwhile directions for future research should include longitudinal studies, embedded calculations of test–retest reliability over short time periods separated by no more than a few weeks, and quantifying a minimum number of trials to sufficiently calculate effect sizes of precision and performance metrics. It is quite possible that given the reproduction format of the continuous recall task, experiments require fewer trials in order to discern accurate outcome measures (since guessing behavior need not be inferred from performance but can be extracted through the reproduced probe itself). Fortunately, these questions of sample size, reliability, and predictive capabilities are not singular to themselves, but go hand in hand with discerning the potential of VWM precision tasks in their capacity to deliver consistently meaningful results that elucidate our understanding of cognitive function.

## Figures and Tables

**Table 1:** Summary of the number of abstracts returned for each of the five databases using the search terminology used in the right-most column.

Database	Number of Abstracts Returned	Search Terminology
Embase	184 abstracts	(EmTREE): these terms were largely unnecessary, as the search was narrowed by topic.
PubMed	151 abstracts	(Visual working memory[Title]) AND ((resolution OR fidelity OR precis *) OR (* OR recall *)) Medical subject heading (MeSH) [short-term] was used (there were no narrower terms).
PSYCHinfo	217 abstracts	(Visual working memory) AND (resolution OR fidelity OR precis *) OR (* OR recall *)
Cochrane Register of Controlled Trials	90 abstracts	We determined the broadest search of relevant articles to be “visual working memory” or VWM
Web of Science	140 abstracts	(“Visual working memory”) Refined by topic: (resolution OR fidelity OR precis *)

The asterisk is used as a wildcard symbol to broaden a search by finding words that start with the same series of letters. Hence, in ‘precis’ it would be looking at ‘precise,’ ‘precision,’ etc.

**Table 2:** Collation of the various study criteria that could affect the interpretation of outcome measures.

Authors	Sample Size	Ages	Healthy/Clinical	Duration b/W Timepoints	Tasks (Trials)	Sequential/Whole Report	Control/Ancillary Tasks (Trials)	Attrition	Primary Outcomes
Zokaei et al. [9]	126 (12 for Parkinson’s longitudinal component)	51–79	Both	3 months	3-item (90) 4-item (200) PD (100–200)	Sequential	Pre-cueing (200; PD patients 100–200) Sensorimotor (25; only completed by 10 healthy older participants. 1-item (200; PD patients 100–200)	None (Only 12 PD patients)	Recall: Precision/Performance
Burnett Heyes et al. [10]	40	7–13	Healthy	2 years	3-item (90)	Sequential	Sensorimotor (25) 1-item (30)	50 *	Recall: Precision/Performance
Fallon et al. [17]	37	Patient:Mean 65 Healthy: Mean 68	Both (20 PD/17 healthy)	1 week to 1 month	Healthy/PD 2-item, 3 sec (64/32) 2-item, 6 sec (64/32)	Whole report	Healthy/PD Update (64/32) Ignore (64/32)	Not reported, presumably none	Recall: Precision (kappa)/Performance
Adam and Vogel [15]	79 (+35 later)	18–35	Healthy	4 months	Orientation (2 * 30))	Whole report	Color-change detection (5 * 30) Visual Search (5 * 48) Anti-Saccade (4 * 36) Raven’s Advanced Progressive matrices (10 min for 18 questions)	7 (and 6 from added 35 member group)	Mean performance (average correct) Change in poor performance

* The 50 attrition number comes from subtracting the 90 student sample at T1 from the 40 student sample at T2.

**Table 3:** Continuous recall.

Author (Year)	Precision Performance	Recall Performance	One-Item Condition
Zokaie [9]	After three months of dopaminergic treatment, precision significantly increased t (11) = 3.01, *p* = 0.012	Significant improvement in performance across all positions F(1,11) = 9.08, *p* = 0.012	No significant difference
Burnett Heyes [10]	T1: 2.33 (1.08) T2: 2.80 (1.19) Student improvement from t1 to t2: (Z = 2.39, *p* = 0.017	Variability around the probed target orientation improved significantly with age, without other sources of error changing t(39) = 3.3, *p* = 0.002	(Z = 2.87, *p* = 0.004; one outlier > 2.5 SD > mean excluded)
Fallon [17]	Trials collapsed across participants on/off medication; no significant difference	Trials collapsed. No significant difference	N/A
others			
**Author (Year)**	**Performance Measure**	**Task Condition**	
Adam and Vogel [15]	Mean performance (average correct)	No improvement in group receiving training: t(47) = −1.68, *p* = 0.1 Improvement in group receiving no training: t(52) = −2.01, *p* = 0.05	
	Change in poor performance	Not calculated	

Summary of the resulting outcome measures of the five studies. While both Zokaie and Burnett Heyes found significant improvements in the three-item task, only Burnett Heyes found significant improvements in the one-item condition. In Fallon, neither of the precision and recall performance measures were significant.

**Table 4:** Summary of the analytical components of the four studies.

Authors (Year)	Effect Sizes (Standardized)	Both Timepoints Reported	Sequential?	Analyses Used	Other
Zokaei et al. [9]	(Sd not provided, unknown if normally distributed)	No	(Individual target values not reported) F(3,33) = 2.5, *p* = 0.07.	*t*-test Mixture Model	Only 12 PD patients were measured at two timepoints. 72 of 126 participants come from Burnett Heyes study. Individual t1/t2 measurements not reported.
Burnett Heyes et al. [10]	0.454	Yes	Yes, improvement on items 1 and 2 but not 3.	Wilcoxon signed-rank *t*-test Mixture Model	All male, prep-school population. Large range, 7–13, for children at key developmental period with a sample too small to separate further by age. We calculated effect size using (Z/√N), where Z is the Z test statistic and N is sample size
Fallon et al. [17]	Fallon determined that difference was not statistically significant for PD patients on/off medication.	Graphed	N/A (whole report)	Mixed-effect model Mixed-anova Wilcoxon signed-rank *t*-test	Very short period between time points (1–4 weeks). One participant could conceivably have 4× as much time between testing as other participants.
Adam and Vogel [15]	No improvement (see this table)	Graphed	N/A (whole report)	Mixed Anova Two-tailed *t*-tests	Focused primarily on motivational factors and effects of feedback on performance.

## Data Availability

In a systematic review, the studies function as the data. We have shared our Zotero folder: https://www.zotero.org/groups/2267504/abstracts_for_review. All of the data from the Adam and Vogel study are publicly accessible at OSF: https://osf.io/839dz/.

## Supplementary Materials

The following supporting information can be downloaded at: https://www.mdpi.com/article/10.3390/vision6010007/s1, Figure S1: Performance Changes Across Time Points; Figure S2: Task Diagrams.
