# Supplementary material for: Systematic Review of the Longitudinal Sensitivity of Precision Tasks in Visual Working Memory
Source: Vision (Basel). 2022 Jan 21;6(1):7. doi: 10.3390/vision6010007 (PMC8883912; doi:10.3390/vision6010007)
Supplement: Supplementary file 1 [file vision-06-00007-s001.zip › vision-1498829-supplementary.pdf]

Supplementary figures

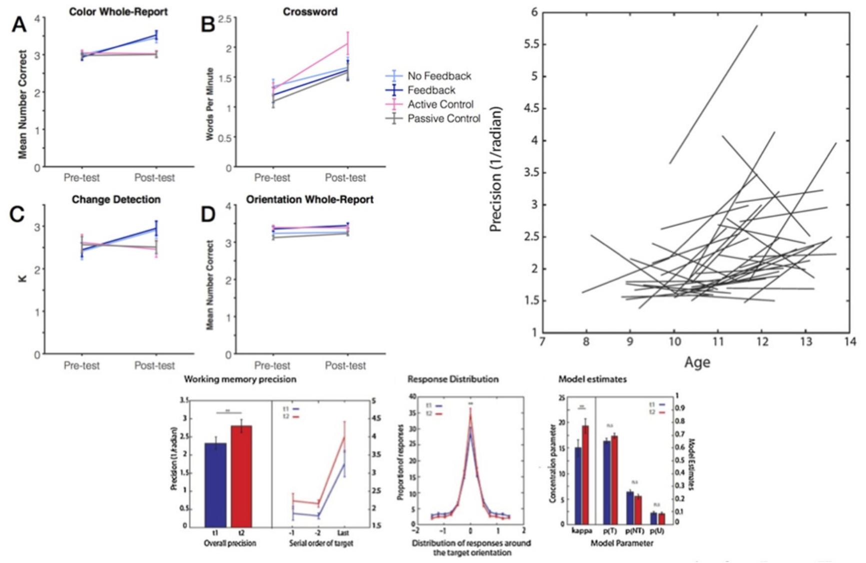

Figure S1: Top right shows the results from Adam and Vogel. Top right is the 3-item performance increase in precision from Burnett Heyes. The bottom three graphs are also from Burnett Heyes and show precision from t1 to t2 (overall and for respective targets, the responses distribution around the target item, and the kappa parameter with a breakdown of the proportion target, non-target and random responses.

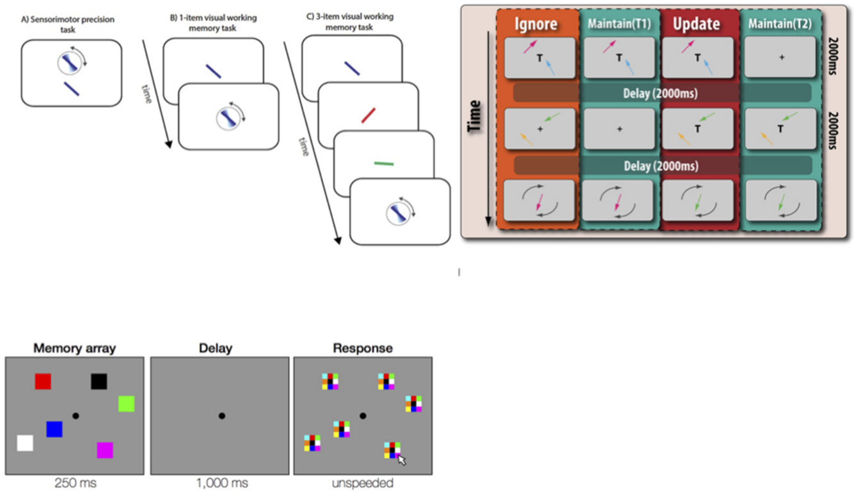

Figure S2: Top left is the sequential continuous recall task (Burnett Heyes and Zokaie). Top right is the whole report continuous recall task (Fallon). Bottom left is the color whole report task (the orientation task was not provided but Adam and Vogel write that “this task was very similar to the color whole report task. Instead of remembering color, participants instead remembered orientations of circles with wedges cut out from them (“wrench-head” stimuli).
